# What’s beyond BRCA Mutational Status in High Grade Serous Ovarian Cancer? The Impact of Hormone Receptor Expression in a Large BRCA-Profiled Ovarian Cancer Patient Series: A Retrospective Cohort Study

**DOI:** 10.3390/cancers14194588

**Published:** 2022-09-22

**Authors:** Emanuele Perrone, Riccardo Tudisco, Pia Clara Pafundi, Davide Guido, Alessandra Ciucci, Enrica Martinelli, Gian Franco Zannoni, Alessia Piermattei, Saveria Spadola, Giulia Ferrante, Claudia Marchetti, Giovanni Scambia, Anna Fagotti, Daniela Gallo

**Affiliations:** 1 Gynecologic Oncology Unit, Department of Woman and Child Health and Public Health, Fondazione Policlinico Universitario A. Gemelli IRCCS, Largo Francesco Vito 1, 00168 Rome, Italy; 2 Universita’ Cattolica del Sacro Cuore, Largo Francesco Vito 1, 00168 Rome, Italy; 3 Epidemiology and Biostatistics Facility Core Research, Gemelli Science and Technology Park, Fondazione Policlinico Universitario A. Gemelli IRCCS, Largo Francesco Vito 1, 00168 Rome, Italy; 4 Bioinformatics Facility Core Research, Gemelli Science and Technology Park (GSTeP) Fondazione Policlinico Universitario A. Gemelli IRCCS, Largo Francesco Vito 1, 00168 Rome, Italy; 5 Unit of Translational Medicine for Woman and Child Health, Department of Woman and Child Health and Public Health, Fondazione Policlinico Universitario A. Gemelli IRCCS, Largo Francesco Vito 1, 00168 Rome, Italy; 6 Gynecopathology and Breast Pathology Unit, Department of Woman and Child Health and Public Health, Fondazione Policlinico Universitario Agostino Gemelli IRCCS, Largo Francesco Vito 1, 00168 Rome, Italy

**Keywords:** estrogen receptors, progesterone receptor, androgen receptor, HGSOC

## Abstract

**Simple Summary:**

Ovarian hormones are involved in ovarian cancer pathogenesis. However, few reports have investigated the hormone receptor pattern according to BRCA mutational status. The aim of this single-center, observational, retrospective study was to explore the relationship between hormone receptor status and BRCA1/2 mutation in a cohort of 207 high-grade serous ovarian carcinoma (HGSOC) patients. Interesting differences emerged between BRCA-mutated and BRCA wild-type women, in terms of pattern of receptor expression and its association to the outcome. On the whole, our findings, though needing further validation, extend our understanding of the complex interplay between BRCA1/2 protein and hormone signaling, suggesting new pathways to be exploited in order to develop future personalized therapy.

**Abstract:**

Several studies have explored the prognostic role of hormone receptor status in high-grade serous ovarian cancer (HGSOC) patients. However, few reports have investigated their expression according to BRCA mutational status. The aim of this single-center, observational, retrospective study was to explore the hormone receptor pattern and its potential prognostic role in a cohort of 207 HGSOC women stratified for BRCA mutational status. To this end, ERα, ERβ1, ERβ2, ERβ5, PR, and AR expression were assessed by immunohistochemistry in 135 BRCA-wild type (BRCA-wt) and 72 BRCA1/2 mutation carriers (BRCA-mut). No significant difference emerged in hormone receptor expression between the two sub-samples, except for a significantly lower ERα expression observed in pre-menopausal BRCA1/2-mut as compared to BRCA-wt patients (*p* = 0.02). None of the examined hormone receptors has revealed a significant prognostic role in the whole sample, apart from the ratio ERα/ERβ5 nuclear, for which higher values disclosed a positive role on the outcome in BRCA-wt subgroup (HR 0.77; CI 0.61–0.96; *p* = 0.019). Conversely, it negatively affected overall survival in the presence of BRCA1/2-mut (HR 1.41; CI 1.06–1.87; *p* = 0.020). Finally, higher PR levels were associated with platinum sensitivity in the whole sample (*p* = 0.019). Our data, though needing further validation, suggest a potential role of oestrogen-mediated pathways in BRCA1/2-associated HGSOC tumorigenesis, thus revealing a possible therapeutic potential for targeting this interaction.

## 1. Introduction

Every year almost 314,000 new ovarian cancer (OC) cases are diagnosed, leading to over 207,000 deaths worldwide [1]. High-grade serous ovarian peritoneal/fallopian-tube cancer (HGSOC) has been estimated to be responsible for 50–60% of all ovarian malignancies and the major cause of all OC-related deaths [2]. Advanced-stage HGSOC 5-year overall survival (OS) still remains poor, usually around 30%, with cytoreductive surgery and DNA-damaging therapy (with/without maintenance therapy, i.e., bevacizumab or poly-adenosine diphosphate ribose polymerase (PARP) inhibitors) as standard of care [3,4]. Typically, beyond 30% of HGSOC tumours are deficient in BRCA1/BRCA2 genes, via either germline or somatic mutations, or hyper-methylation [5].

Ovarian hormones, including estrogen, androgen, and progesterone, are systemically and locally involved in OC pathogenesis [6]. Oestrogens have long been considered among the effective OC triggers, acting via estrogen receptors (ERs), although their real impact and mechanistic details still remain unclear [6,7]. Estrogen signaling is the result of a balance between two opposing forces, i.e., two distinct receptors (ERα and ERβ) and their splice variants [7]. ERα and ERβ are members of the nuclear receptor superfamily of ligand-dependent transcription factors and share both structural and functional homologies, though encoded by separate genes. In the presence of ligands, ERα and ERβ bind to the estrogen responsive element (ERE) located in gene promoter regions, either as homodimers (ERα/ERα or ERβ/ERβ) or heterodimers (ERα/ERβ), to regulate target genes’ transcriptional activity. Several ERβ isoforms have been so far reported: wild-type ERβ (ERβ1) encodes the full-length, 530-amino-acid receptor protein and is the only fully functional isoform able to bind ligands; ERβ2 to ERβ5, which use alternative exons, instead encode for variant receptors with different C-termini, and may modulate estrogen action when dimerized with either ERβ1 or ERα [8]. ERα is considered responsible for enhanced cancer-cell proliferation, whereas an anti-proliferative and pro-apoptotic effect of ERβ1 has been shown [7]. ERα and ERβ isoforms are expressed in most HGSOC, though without definitive data on their prognostic/predictive role in the disease [9,10,11,12].

High androgen levels have also been associated with an increased risk of OC initiation, and literature data have suggested that androgen receptor (AR) signaling might play an important role in cancer growth [13]). AR positivity rate in HGSOC is around 30% [14], though with a reported wide range (10-to-68%), and a still-controversial prognostic role [13]. On the other hand, clinical and epidemiological data suggest a potential protective role of progesterone against ovarian carcinogenesis [15]. The biological response to progesterone is mediated by three isoforms of progesterone receptor (PR): full-length PRB, N-terminally truncated PRA, and non-functional PRC. PRB and PRA act as ligand-activated transcription factors, whereas PRC may serve to sequester the ligand, as it is unable to bind DNA [15]. Around 30–50% of HGSOC patients are PR-positive and a strong PR expression has been considered a favourable prognostic marker in HGSOC [9,10].

Notably, there is evidence of a strong regulatory interplay between BRCA1/2 and steroid hormone action. In fact, few reports have shown that BRCA1/BRCA2 mutations carriers are exposed to higher titres of estradiol and progesterone [16]. Additionally, there are data on BRCA1 protein interaction with ERα and AR leading to ERα inhibition and the stimulation of AR activity [17]. Nevertheless, few reports have fully investigated steroid hormone receptor expression in BRCA1/2-mutated and in sporadic HGSOC, and their role as prognostic biomarkers in different populations [18]. Thus, we sought to explore the hormone receptor profile and its potential prognostic impact in a well-characterized cohort of HGSOC patients stratified for non-BRCA (BRCA-wt) to BRCA1/2 mutation carriers (BRCA1\2-mut).

## 2. Materials and Methods

### 2.1. Study Design and Participants

In this single-centre observational retrospective cohort study, we enrolled HGSOC women admitted to the gynaecological oncology unit of Policlinico Universitario “A. Gemelli” IRCCS (Rome, Italy) between 2014 and 2019, with known BRCA-1/2 germline/somatic mutation status and available histopathologic and molecular features. The unavailability of paraffin-embedded samples for histological analyses and the lack of written informed consent were specific exclusion criteria. Histopathologic features and epidemiologic, clinical, and surgical data were reviewed and collected in an electronic database (Appendix A.1). The study protocol was approved by our local ethics committee, in accordance with 1976 Declaration of Helsinki and its later amendments (N° Prot. DIPUSVSP-26-05-2070, Prot. ID 3257).

### 2.2. Immunohistochemistry

Immunohistochemical staining was performed as previously described [9,11,19], either manually or in a Dako AutoStainer (Appendix A.2).

### 2.3. Evaluation of Immunohistochemical Staining

Hormone receptor scoring was assessed as previously reported [11,19]. Briefly, the mean percentage of stained cells was classified as follows: 0 = negative, 1 = 1–10%, 2 = 11–33%, 3 = 34–66%, 4 = 67–100%. Staining intensity was graded from 1 to 3 (1-weak staining, 2-moderate staining and 3-strong staining). The two obtained values were multiplied to calculate an immune-reactive score (IRS, maximum value 12). Two investigators (GFZ and SS) carried out the assessment.

### 2.4. Outcomes

The primary endpoint was to describe a potential association between BRCA status and hormone receptor profile in HGSOC women. We further assessed OS across BRCA mutational status, as well as potential predictors of both OS and platinum resistance. Patients were stratified as BRCA wild-type or mutated (BRCA1/2).

### 2.5. Statistical Analysis

We enrolled 207 women, 65.2% of whom were BRCA-wt. Given the retrospective study design, no prior sample-size calculation was available. However, such sample size is able to achieve an 80% power to detect a difference of 0.4 using a two-sided Mann–Whitney U test assuming a normal data distribution, a 5% significance level, and standard deviation (SD) of 1.0 in both groups. Power analysis was conducted with PASS2021 [20].

All the data were preliminary summarized by descriptive statistics, both overall and according to BRCA mutation status (wild-type vs. BRCA1/2-mut). Qualitative data were described as absolute and relative percentage frequency, whilst quantitative as mean (±SD) or median and interquartile range (IQR). Gaussian distribution of quantitative variables was assessed by the Shapiro–Wilk test. Between-groups differences on qualitative data were computed by either chi-square test or Fisher–Freeman–Halton’s exact test. Quantitative variables were instead assessed either by Student’s *t* test or Mann–Witney U test. Missing values in quantitative variables, all <5%, were treated by multiple imputation with lasso regression methods centred on the mean by using *imputeR* R package [21]. Differences across BRCA mutational status, classified as “wild-type”, “BRCA1”, and “BRCA2” mutated, stratified for menopause status, were assessed by the Kruskal–Wallis test. Pairwise comparisons were assessed by the Dunn’s test, with false discovery rate correction for multiple comparisons. All data were further presented by “violin plots” drawn with R packages “*ggpubr*”, “*ggplot2*”, and “*ggstatsplot*”.

The raw effects of each hormone receptor expression (HRE) and clinical data (predictor) on OS were assessed by ordinary proportional hazard Cox models, reporting hazard ratios (HRs) and 95% confidence intervals (CIs). To evaluate the combined effects between HREs/clinical predictors and BRCA mutations, multivariable age-adjusted interaction Cox models were fitted, one for each predictor, and the related interaction HRs (IHR) reported.

Potential predictors of platinum resistance were instead assessed by logistic regression models. To evaluate the combined effects between HERs/clinical data and BRCA mutations, multivariable interaction models were fitted, one per each predictor, reporting the interaction odd ratios (IOR) (Appendix A.3).

Multivariable interaction models were applied in place of classic multivariable models in order to better assess the role of each predictor on clinical outcomes according to BRCA mutational status.

Statistical significance was set at *p*-value < 0.05. *p*-values between 0.05 and 0.10 were also reported as suggestive. All analyses were performed by R software version 4.2.0 (CRAN^®^, R Core Team, 2022, Vienna, Austria) [22], and its packages *Hmisc*, *survival*, *survminer*, and *coxphw*.

## 3. Results

### 3.1. Patient Features

Two hundred and seven women were included in the study, 135 BRCA-wt and 72 BRCA1\2-mut (45 BRCA1 and 27 BRCA2). Overall mean age was equal to 59.1 ± 11.4 years, consistent with previous data [10], with BRCA-wt significantly older than BRCA-mut patients (60.6 ± 11.5 yrs. vs. 56.4 ± 10.8 yrs., *p* = 0.011), with over 70% of patients in a menopausal status (76.3% vs. 63.9%, *p* = 0.058). Median BMI (24 kg/m^2^, IQR 21.5–27.7) was similar between the two sub-samples. Moreover, interval debulking surgery (IDS) as primary treatment was much more prominent in BRCA1/2 patients (37.5% vs. 26.7%, *p* = 0.034). PARP-inhibitor therapy was administered only in a small subset of BRCA-mut patients, whilst bevacizumab did not significantly differ between BRCA statuses.

Overall mortality rate was 34.8% and relapse rate 73%, significantly higher among BRCA-wt women (respectively, 43% vs. 19.4% in BRCA1/2; *p* = 0.001 and 77.8% vs. 63.9%; *p* = 0.048). As well, BRCA-wt patients developed a significantly higher rate of platinum resistance (37.8% vs. 13.9% in BRCA1/2-mut; *p* < 0.001) (Table 1).

### 3.2. Hormone Receptor Status in HGSOC

**Figure 1:**
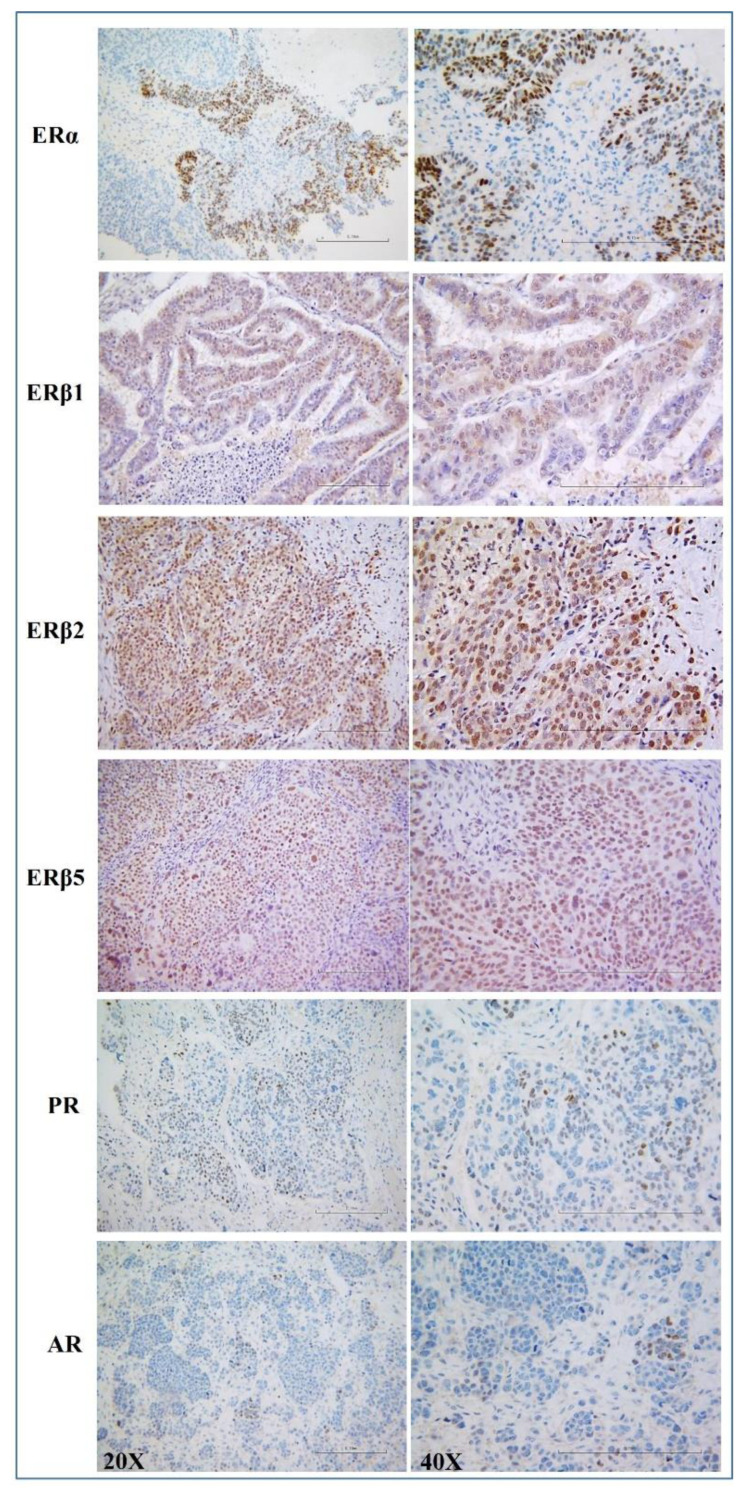
Immunohistochemical analysis of six hormone receptors in primary high-grade serous ovarian cancer (HGSOC). Representative pictures for ERα, ERβ1, ERβ2, ERβ5, PR, and AR immunostaining in HGSOC patients (magnification 20×, 40×), displaying both nuclear and cytoplasmic protein expressions.

Figure 1 shows representative pictures for ERα, ERβ1, ERβ2, ERβ5, PR, and AR. On a patient level, considering a cut-off hormone receptor expression levels of >10%, nuclear ERα was expressed in 78%, ERβ1 in 92%, ERβ2 in 97%, ERβ5 in 96%, PR in 30%, and AR in 29%. Cytoplasmic reaction was also evident for ERβ1, ERβ2, and ERβ5 in about 64%, 59%, and 29% of cases, respectively.

Table 2 shows the median hormone receptor histoscores in the overall sample. No significant difference emerged across BRCA mutational status, except for a suggestive association towards a higher ERα score among BRCA-wt patients (median 4 (IQR 2–8) vs. 3 (IQR 2–6) in BRCA1/2-mut, *p* = 0.090) (Table 2).

**Figure 2:**
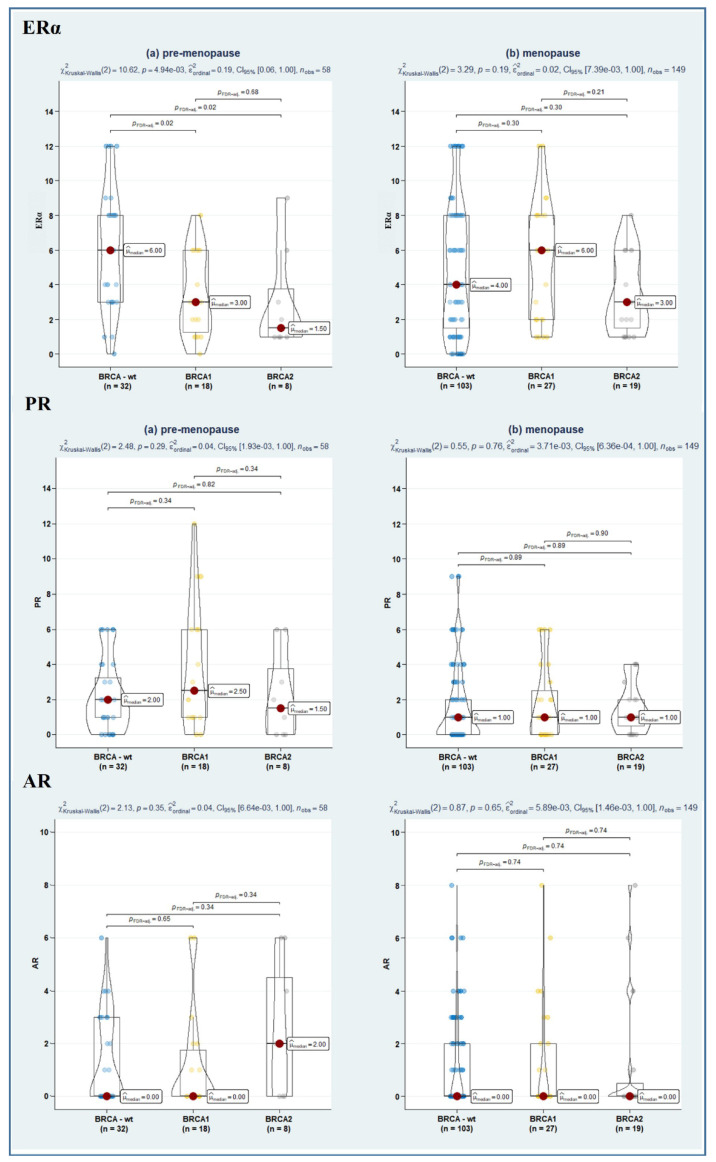
Violin plots depicting the relationship between ERα (**upper panel**), PR (**in the middle**), and AR (**lower panel**) in BRCA-wt, BRCA1, and BRCA2 mutated women according to menopausal status. Both overall and pairwise comparisons are reported, with FdR correction. Blue, yellow and grey dot respectively represents BRCA-wt, BRCA1 and BRCA2 patients pertaining that score of molecular marker.

**Figure 3:**
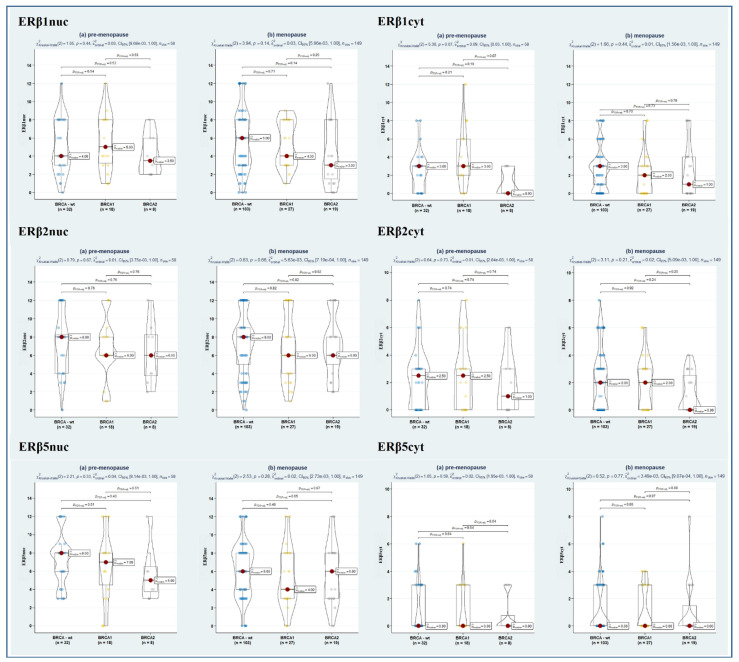
Violin plots depicting the relationship between ERβ1nuc (**upper left panel**), ERβ1cyt (**upper right panel**), ERβ2nuc (**middle left panel**), ERβ2cyt (**middle right panel**), ERβ5nuc (**lower left panel**), and ERβ5cyt (**lower right panel**) in BRCA-wt, BRCA1, and BRCA2 mutated women according to menopausal status. Both overall and pairwise comparisons are reported, with FdR correction. Blue, yellow and grey dot respectively represents BRCA-wt, BRCA1 and BRCA2 patients pertaining that score of molecular marker.

**Figure 4:**
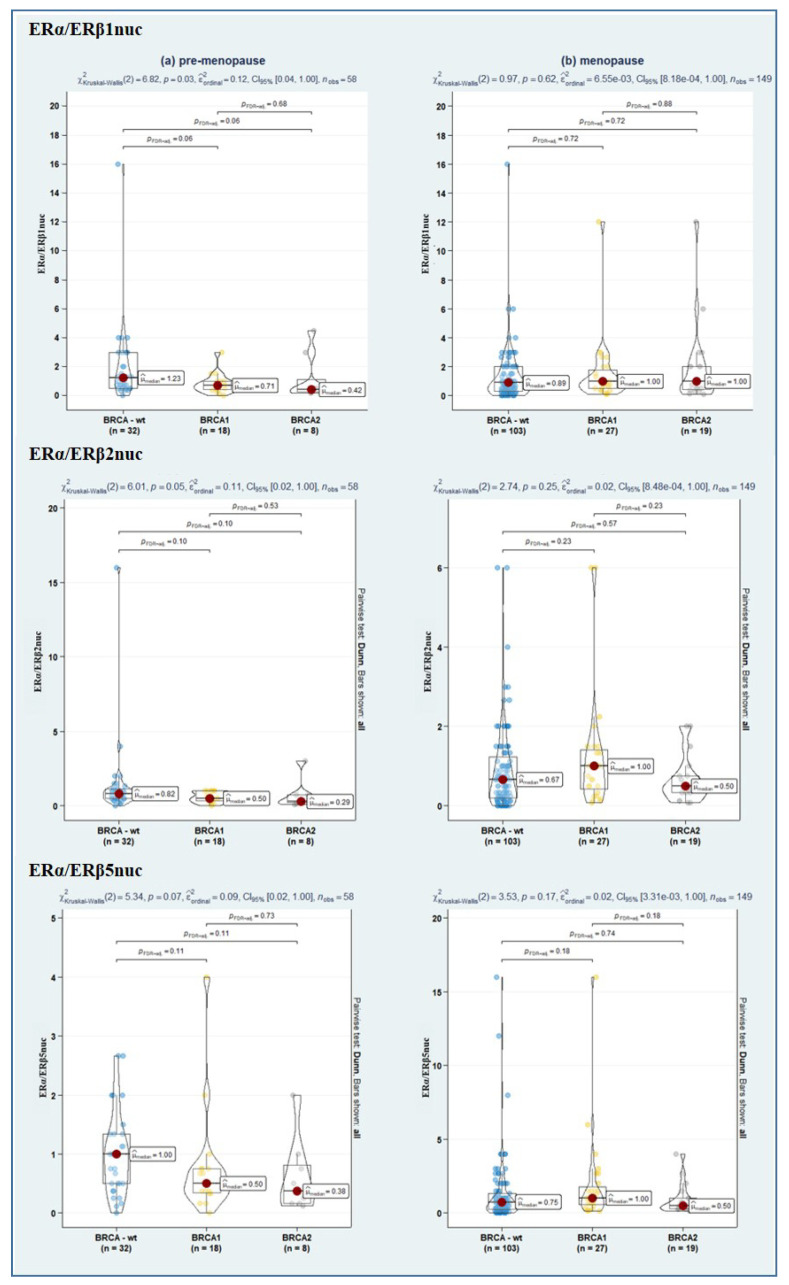
Violin plots depicting the relationship between ERα/ERβ1nuc (**upper panel**), ERα/ERβ2nuc (**in the middle**), and ERα/ERβ5nuc ratio (**lower panel**) in BRCA-wt, BRCA1, and BRCA2 mutated women according to menopausal status. Both overall and pairwise comparisons are reported, with FdR correction. Blue, yellow and grey dot respectively represents BRCA-wt, BRCA1 and BRCA2 patients pertaining that score of molecular marker.

Since emerging evidence suggests that the ERα/ERβ ratio is likely more useful than single-receptor evaluation [23]; we also assessed the relative level of nuclear ER subtype-specific expression (in terms of the ratio of ERα/ERβ1, ERα/ERβ2, and ERα/ERβ5), though no significant difference emerged among the study sub-samples (Table 2). Instead, remarkably, stratification of hormone receptor expression according to both BRCA and menopausal status revealed a significantly lower ERα expression in BRCA1- and BRCA2-mutated as compared to BRCA-wt (*p* = 0.02, in both cases). As well, a further suggestive association towards a lower ERα/ERβ1 ratio in BRCA1/2-mut-carrier tumours was observed (*p* = 0.06, in both cases) (Figure 2, Figure 3 and Figure 4).

### 3.3. Assessment of Potential Predictors of Overall Survival across BRCA Mutational Status

In the whole sample, the ordinary Cox regression models confirmed the well-known favourable prognostic role of BRCA1/2 mutation on OS (HR: 0.34, 95%CI 0.18–0.61; *p* < 0.001), as well as primary treatment, both PDS (HR: 0.06, 95%CI 0.03–0.11; *p* < 0.001) and IDS (HR: 0.10, 95%CI 0.05–0.20; *p* < 0.001). Conversely, menopausal status (HR: 2.32, 95%CI 1.24–4.31; *p* = 0.008), ascites (HR: 2.35, 95%CI 1.42–3.88; *p* = 0.001) and an RT > 10 mm (HR: 7.50, 95%CI 4.23–13.28; *p* < 0.001) negatively affected OS. Univariable analysis instead did not disclose any significant role of hormone receptor markers, except for a suggestive protective role of PR expression (HR: 0.90, 95%CI 0.80–1.01; *p* = 0.067) (Table 3).

As for the predictors’ main effects, in age-adjusted interaction Cox models (i.e., within wild-type BRCA condition), both PDS (HR: 0.09, 95%CI 0.04–0.18; *p* < 0.001) and IDS (HR: 0.14, 95%CI 0.06–0.29; *p* < 0.001) confirmed their positive effect on OS. Conversely, an RT > 10 mm (HR: 6.26, 95%CI 3.31–11.83; *p* < 0.001) was confirmed as a negative prognostic factor. Notably, among molecular markers, a higher ERα/ERβ5nuc ratio instead positively affected OS within wild-type BRCA condition (HR: 0.77, 95%CI 0.61–0.96; *p* = 0.019). Conversely, the interaction between the ERα/ERβ5nuc ratio and presence of BRCA1/2 mutation disclosed a negative prognostic role on OS in this specific subset of patients (IHR: 1.41, 95%CI 1.06–1.87; *p* = 0.020) (Table 3).

### 3.4. Assessment of Potential Predictors of Platinum Resistance across BRCA Mutational Status

We further assessed potential predictors of platinum resistance. At univariable analysis, logistic regression models confirmed the well-known predictive role of BRCA1/2 mutation (OR: 0.27, 95%CI 0.13–0.56; *p* = 0.001), cytoreductive surgery (PDS—OR: 0.01, 95%CI 0.00–0.10; *p* < 0.001) and IDS (OR: 0.03, 95%CI 0.00–0.21; *p* = 0.001). Conversely, menopause (OR: 2.16, 95%CI 1.03–4.52; *p* = 0.041) and ascites (OR: 2.47, 95%CI 1.31–4.64; *p* = 0.005), alongside with an RT > 10 mm (OR: 10.88, 95%CI 4.02–29.50; *p* < 0.001), revealed significantly involved in platinum-resistance onset. Among hormone receptors, instead, a higher PR score was significantly associated with a lower platinum resistance (OR: 0.83, 95%CI 0.71–0.97; *p* = 0.019). Besides this, no association emerged for the interaction effects between predictors and BRCA status (Table 4).

## 4. Discussion

Few studies have compared steroid hormone receptors’ profile in hereditary and sporadic OC. Here we assessed ERα, ERβ1, ERβ2, ERβ5, PR, and AR expression in a large retrospective HGSOC-patient cohort in order to investigate a potential association between BRCA status and hormone receptors. Our findings disclosed the expression of ERα and the ERβ variants ERβ1, ERβ2, and ERβ5 in most of tumours, whilst only a limited positivity emerged for PR and AR, consistent with previous data from us and other groups on hormone receptor status in HGSOCs [9,10,11,12,13,24]. Notably, no significant difference emerged in the individual steroid receptor expression between BRCA1/2-associated and sporadic HGSOCs, despite a suggestive lower ERα expression in BRCA1/2-mut vs. BRCA-wt. These data are consistent with those from Aghmesheh and colleagues in 44 epithelial-OC [18]. However, interestingly, stratification according to both BRCA and menopausal status revealed a significant difference in ERα expression, as compared to premenopausal BRCA-wt, with a lower receptor score in premenopausal BRCA1- and BRCA2-mutation. These findings are partially consistent with those reported in hereditary, BRCA1-associated breast cancer (BC), ERα-negative in ≈90% of cases [25] (reviewed by [26]), while BRCA2-mutated patients show a distribution of ER staining-like controls [25]. A potential explanation might lie in the commonly reduced expression of BRCA1 protein in sporadic OC, alongside its large occurrence through mechanisms other than somatic mutation [27]. Overall, our findings thus suggest that main differences might occur between two hormonally regulated tissues, such as breast and ovary, on the regulatory interplay between BRCA1 and ERα.

Remarkably, we further displayed the ERα/ERβ5 ratio’s opposite role as prognostic factor for OS in wt- and mut-BRCA1/2 patients. Indeed, we found that a higher ERα/ERβ5 expression was associated with a longer survival among BRCA-wt patients, whilst in BRCA1/2-mutated women a negative prognostic role emerged. Moreover, a subgroup analysis according to either BRCA1 or BRCA2 status showed that the interaction between the ERα/ERβ5 ratio, alongside BRCA1 mutation, portends a negative prognostic role in OS (IHR 1.59, 95%CI 1.25–2.04; *p* < 0.001), whilst in BRCA2-mutated the behaviour was similar to BRCA-wt women (IHR 0.50, 95%CI 0.16–1.56; *p* = 0.234). Further studies are needed to clarify the biological/pathological mechanisms underpinning this relationship. Of note, in different human cancer cells, BRCA1 globally represses ERα activity [28] (reviewed by [26]). Likewise, BRCA1 BC-associated mutations either abolish or reduce its ability to inhibit ERα activity [26]. Therefore, we would expect that the direct role played by BRCA1 in the control of ERα-mediated transcription reduces oestrogen’s effects on proliferation, angiogenesis, and TME-mediated tumour growth. This might be particularly relevant when considering that: (a) high E2 levels are often observed in OC patients/tissues (reviewed by [29,30]), and (b) carriers of BRCA1/2 mutations have increased oestrogen levels [16].

With regard to ERβ5, it is expressed at high levels in the human ovary [31], as well as in OC, as demonstrated in our study, consistently with previous findings [11,12]. According to previous literature, ERβ5 owns ligand-independent transcriptional properties and ERα-modulating activities [32,33]. Notably, an ERβ5 oncogenic role has been reported in epithelial-OC, which occurs through the regulation of cell migration, invasion, and proliferation [12]. As for the ERβ5-mediated ERα-modulating activity, context-dependent cell effects have been reported in the outcomes of ERα/ERβ5 heterodimers in epithelial cells. Indeed, Collins et al. have recently demonstrated an increased oestrogen responsiveness of ERα+ Ishikawa cells by ERβ5 [33]. Conversely, previous reports showed that ERβ5 can inhibit ERα-dependent activation of an ERE reporter gene in COS7 cells [32,34]. Thus, even considering the oncogenic properties reported for ERβ5 in OC, we might speculate that in BRCA-wt patients, BRCA1 inhibition of ERα signalling plays a major role over that potentially exerted by the reduced oestrogen responsiveness following ERα/ERβ5 heterodimerization. Besides this, an inhibition of the oestrogen-independent transcriptional activity of ERβ5 by ERα has been reported [32], i.e., high ERα levels efficiently control ERβ5-mediated transcriptional activity. On the other hand, in BRCA1-mut women, lacking BRCA1-mediated repression of ERα transcriptional activity, high ERα levels, not inhibited by ERβ5, result in increased ligand-dependent transcriptional activity which may, in turn, stimulate tumour growth.

Notably, clinical studies have suggested that endocrine therapy (letrozole or tamoxifene) may represent a reasonable treatment option for patients with ERα-positive HGSOC (reviewed by [35]). However, an ongoing challenge is to identify those patients who will really benefit from these treatments. In this context, controversial data have been reported about the association between clinical response and ERα expression, with several factors possibly accounting for the observed discrepancy (reviewed by [35]). Overall, ERα expression by itself seems insufficient to recognize which tumours are under oestrogen growth control. In light of this, our findings, if confirmed, could set the stage for future translational trials, aimed to better identify oestrogen-responsive HGSOCs.

Interestingly, we further observed a PR expression suggestive of a favourable survival of HGSOC and indicative of platinum sensitivity as well, consistent with previous literature [10,36]. Likewise, the observed lack of association between ERα levels and survival in HGSOC patients observed in our study is consistent with a large study conducted by the Ovarian Tumour Tissue Analysis consortium of 1742 HGSOCs [10]. Beyond this, further data reported in the meta-analysis by Shen and colleagues showed an association between ERα expression and a better OS in unclassified epithelial OC, though not related to outcome in the serous type [37]. Finally, our findings failed to confirm the prognostic value of cytoplasmic ERβ2 previously observed in a small cohort of advanced serous OC patients [11]. This could be related to diverse factors, including differences in therapeutic approaches between the two series examined (i.e., use of bevacizumab or PARP-inhibitors in >40% of patients in the present study), or differences in tissue processing, which may have significantly affected immunohistochemical results [38].

Some study limitations need to be acknowledged. First, the retrospective design affected sample size determination, which was based on a post-hoc calculation and did not allow us to select homogeneous sub-cohorts for each BRCA mutational status. As such, we could not investigate in depth the stratification across BRCA1 and BRCA2 mutations and their further sub-analysis for menopausal status, which could have been affected by a power bias. In addition, even though low, PR and AR levels seemed localized in the nucleus, being potentially active. It would have been worthy to assess ratios between nuclear ERs and PR or AR, to provide further insights in their molecular role, especially considering the pre- vs. post-menopausal status. However, we could not provide such analysis in our sample, due to the extremely high percentage of null scores of PR and AR, denominators of the ratios. The potential use of pseudocounts to make all observed counts strictly positive, actually already applied for ERα/ERβs ratios, was in that case possible due to the presence of <5% null score values. Such a high percentage of null score, i.e., the large asymmetry of AR and PR scores distribution, instead did not allow for the use of arbitrary pseudocounts, which would have provided dramatically biased results [39].

Nevertheless, this study has several strengths. First, few studies have investigated the relationship between BRCA1/2 and steroid hormone receptor status in large HGSOC series. Moreover, despite the retrospective design and the expected selection bias, the reported findings on clinical outcomes uniquely confirmed the validity of our series. As well, the use of multivariable interaction Cox/logistic regression models in place of classic multivariable models allowed us to better assess the role of each predictor on clinical outcomes according to BRCA mutational status.

## 5. Conclusions

In conclusion, this study extends our understanding of the complex interplay between BRCA1/2 protein and hormone signalling, thus suggesting a potential role of oestrogen-mediated pathways in BRCA1/2-associated HGSOC tumorigenesis. Undoubtedly, to legitimate our hypotheses and improve the potential prognostic/predictive role of steroid hormone receptors according to BRCA and menopausal status, further large-scale prospective studies are needed. Nonetheless, our assumptions may represent the first step to determine and investigate the molecular interaction between two crucial players of OC pathogenesis, in order to potentially develop future therapeutic targets.

## Figures and Tables

**Table 1:** General characteristics of the study sample according to BRCA-wt and BRCA1/2-mut (*n* = 207) *.

	BRCA Mutation			
	Overall (*n* = 207)	wt-BRCA (*n* = 135)	BRCA1/2 (*n* = 72)	*p* **
**Age (yrs.)**	59.1 (11.4)	60.6 (11.5)	56.4 (10.8)	**0.011**
**Baseline BMI (kg/m^2^)**	24 (21.6–27.7)	24.1 (21.9–27.7)	24.0 (20.6–27.8)	0.532
**Menopause, No. (%)**	149 (72.0)	103 (76.3)	46 (63.9)	*0.058*
**Ca125 at diagnosis**	880.2 (318–2136.1)	1003 (341.5–2237.8)	601 (285–1762)	*0.080*
**Ascites, No. (%)**				0.445
*Yes*	111 (53.6)	75 (55.6)	36 (50.0)	
*No*	96 (46.4)	60 (44.4)	36 (50.0)	
**FIGO Stage, No. (%)**				0.519
*I–II*	4 (1.9)	2 (1.5)	2 (2.8)	
*III–IV*	203 (98.1)	133 (98.5)	70 (97.2)	
**Primary Treatment, No. (%)**				**0.034**
*PDS*	125 (60.4)	82 (60.7)	43 (59.7)	
*IDS*	63 (30.4)	36 (26.7)	27 (37.5)	
*Non cytoreduced*	19 (9.2)	17 (12.6)	2 (2.8)	
**RT, No. (%)**				*0.079*
*0*	162 (78.3)	104 (77.0)	58 (80.6)	
*1–10 mm*	21 (10.1)	11 (8.2)	10 (13.9)	
*>10 mm*	24 (11.6)	20 (14.8)	4 (5.6)	
**Therapy, No. (%)**				
PARP-i	8 (3.9)	-	8 (11.1)	**<0.001**
Bevacizumab	87 (42.0)	52 (38.5)	35 (48.6)	0.184
**Outcomes**				
Overall Survival, No. (%)	135 (65.2)	77 (57.0)	58 (80.6)	**0.001**
OS follow-up (months)	34 (22–43)	33 (19–42)	38 (32–44.5)	**0.001**
Relapse, No. (%)	151 (73.0)	105 (77.8)	46 (63.9)	**0.048**
Platinum resistance, No. (%)	61 (29.5)	51 (37.8)	10 (13.9)	**<0.001**

**Abbreviations:** BMI: body mass index; FIGO: International Federation of Gynaecology and Obstetrics; IDS: interval debulking surgery; PDS: primary debulking surgery; RT: residual tumour; PARP-i: poly adenosine diphosphate-ribose polymerase inhibitors; OS: overall survival. * Descriptive statistics are expressed as median (interquartile range) or mean (standard deviation) for quantitative variables, as absolute and percentage frequencies for qualitative variables. ** *p*-values were computed, as for qualitative variables, by the chi-square test or the Fisher–Freeman–Halton’s exact test. For quantitative variables, Student’s *t* test (if normally distributed) or Mann–Whitney U test were applied. In **bold**: the significant results (*p* < 0.05), in *italics*: the suggestive results (0.05 < *p* < 0.10).

**Table 2:** Molecular characteristics of the study sample according to BRCA-wt and BRCA/1–2 (*n* = 207) *.

	BRCA Mutation			
	Overall (*n* = 207)	wt-BRCA (*n* = 135)	BRCA1/2 (*n* = 72)	*p* **
AR score	0 (0–2)	0 (0–2)	0 (0–2)	0.711
PR score	1 (0–3)	1 (0–3)	1 (0–4)	0.157
ERα score	4 (2–8)	4 (2–8)	3 (2–6)	*0.090*
Nucleus ERβ1 score	4 (3–8)	4 (3–8)	4 (3–8)	0.218
Cytoplasm ERβ1 score	3 (0–3)	3 (1–3)	2 (0–3)	0.425
Nucleus ERβ2 score	8 (4–9)	8 (4–9)	6 (4–8)	0.227
Cytoplasm ERβ2 score	2 (0–3)	2 (0–3)	2 (0–3)	0.400
Nucleus ERβ5 score	6 (4–8)	6 (4–8)	6 (3–8)	*0.097*
Cytoplasm ERβ5 score	0 (0–3)	0 (0–3)	0 (0–3)	0.992
ERα/ERβ1nuc ratio	0.8 (0.4–2.0)	1.0 (0.4–2.0)	0.8 (0.4–1.8)	0.603
ERα/ERβ2nuc ratio	0.7 (0.3–1.0)	0.7 (0.3–1.1)	0.5 (0.3–1.0)	0.603
ERα/ERβ5nuc ratio	0.8 (0.3–1.3)	0.8 (0.3–1.3)	0.7 (0.3–1.4)	0.892
**P53 Status**				0.763
*Wild-type*	11 (5.3)	8 (5.9)	3 (4.2)	
*Mutated null-type*	55 (26.6)	34 (25.2)	21 (29.2)	
*Mutated overexpressed*	141 (68.1)	93 (68.9)	48 (66.7)	

**Abbreviations**: wt: wild-type; AR: androgen receptor; PR: progesterone receptor; ER: oestrogen receptor; * Descriptive statistics are expressed as median (interquartile range) for quantitative variables, as absolute and percentage frequencies for qualitative variables. ** *p*-values were computed, as for qualitative variables by the Fisher–Freeman–Halton’s exact test. For quantitative variables, Mann–Whitney U test was applied. In **bold**: the significant results (*p* < 0.05), in *italics*: the suggestive results (0.05 < *p* < 0.10).

**Table 3:** Survival Analysis on BRCA-wt vs. BRCA mutated (*n* = 207) *.

	Ordinary Cox Model	Interaction Cox Model	
		Predictor Main Effect (with BRCA = 0 [wt])	Predictor × BRCA Interaction
*Death (Primary Outcome)*	HR (95% CI); *p*	HR (95% CI); *p*	IHR (95% CI); *p*
BRCA (Ref. = wt)	**0.34 (0.18; 0.61); <0.001**	-	-
BMI at baseline	1.01 (0.97; 1.06); 0.637	1.01 (0.95; 1.07); 0.855	0.98 (0.89; 1.07); 0.609
Menopause	**2.32 (1.24; 4.31); 0.008**	0.71 (0.29; 1.69); 0.438	2.34 (0.45; 12.23); 0.315
Ca125	1.00 (1.00; 1.00); 0.201	1.00 (1.00; 1.00); 0.317	1.00 (0.99; 1.00); 0.660
Ascites	**2.35 (1.42; 3.88); 0.001**	**1.73 (1.00; 2.98); 0.049**	3.20 (0.65; 15.77); 0.152
Primary treatment (*Ref. Non cytoreduced*)			
* PDS*	**0.06 (0.03; 0.11); <0.001**	**0.09 (0.04; 0.18); <0.001**	**0.09 (0.02; 0.41); 0.002**
* IDS*	**0.10 (0.05; 0.20); <0.001**	**0.14 (0.06; 0.29); <0.001**	*0.32 (0.10; 1.03); 0.055*
RT (ref = 0)			
* 1–10 mm*	1.32 (0.60; 2.94); 0.488	1.86 (0.78; 4.42); 0.160	0.28 (0.03; 2.64); 0.268
* >10 mm*	**7.50 (4.23; 13.28); <0.001**	**6.26 (3.31; 11.83); <0.001**	0.88 (0.17; 4.51); 0.880
**Molecular markers**			
Nucleus AR score	0.93 (0.82; 1.06); 0.303	0.90 (0.77; 1.06); 0.202	1.12 (0.84; 1.50); 0.433
PR score	*0.90 (0.80; 1.01); 0.067*	0.95 (0.84; 1.08); 0.460	0.88 (0.64; 1.21); 0.441
ERα score	0.99 (0.93; 1.06); 0.840	0.95 (0.88; 1.02); 0.175	1.13 (0.93; 1.36); 0.211
Nucleus ERβ1 score	1.05 (0.98; 1.14); 0.177	1.02 (0.94; 1.11); 0.413	1.05 (0.86; 1.29); 0.497
Cytoplasm ERβ1 score	1.00 (0.91; 1.10); 0.935	0.97 (0.86; 1.09); 0.642	1.12 (0.91; 1.39); 0.289
Nucleus ERβ2 score	1.01 (0.94; 1.08); 0.832	0.97 (0.90; 1.06); 0.534	1.05 (0.88; 1.25); 0.587
Cytoplasm ERβ2 score	1.04 (0.94; 1.16); 0.441	1.02 (0.91; 1.15); 0.696	1.04 (0.78; 1.39); 0.762
Nucleus ERβ5 score	0.99 (0.92; 1.07); 0.794	0.99 (0.90; 1.08); 0.803	0.96 (0.78; 1.17); 0.669
Cytoplasm ERβ5 score	0.89 (0.77; 1.03); 0.129	0.94 (0.81; 1.10); 0.438	0.82 (0.50; 1.33); 0.418
ERα/ERβ1nuc ratio	0.91 (0.78; 1.06); 0.215	0.89 (0.74; 1.08); 0.239	1.02 (0.69; 1.49); 0.934
ERα/ERβ2nuc ratio	0.92 (0.75; 1.12); 0.396	0.85 (0.65; 1.12); 0.248	1.29 (0.79; 2.09); 0.306
ERα/ERβ5nuc ratio	0.97 (0.85; 1.11); 0.714	**0.77 (0.61; 0.96); 0.019**	**1.41 (1.06; 1.87); 0.020**
P53 Status (Ref. wt)			
*Mutated null-type*	1.30 (0.39; 4.40); 0.667	1.68 (0.49; 5.77); 0.410	Inf^ (0.00; Inf^); 0.996
*Mutated overexpressed*	1.26 (0.39; 4.01); 0.695	1.23 (0.38; 3.98); 0.733	Inf^ (0.00; Inf^); 0.996

**Abbreviations:** wt: wild type; BMI: body mass index; IDS: interval debulking surgery; PDS: primary debulking surgery; RT: residual tumour; AR: androgen receptor; PR: progesterone receptor; ER: oestrogen receptor; HR: hazard ratio; IHR: interaction hazard ratio; 95% CI: 95% confidence interval; Ref.: reference; ^Inf: infinite (due to poor or null variability within predictors) * In **bold**: the significant results (*p* < 0.05), in *italics*: the suggestive results (0.05 < *p* < 0.10).

**Table 4:** Logistic Regression on BRCA-wt vs. BRCA mutated (*n* = 207) *.

	Univariable Analysis			Interaction Multivariable Model	
	Platinum Resistance			Predictor Main Effect (with BRCA = 0 [wt])	Predictor x BRCA Interaction
	Yes (*n* = 61)	No (*n* = 146)	OR (95% CI); *p*	OR (95% CI); *p*	IOR (95% CI); *p*
Age	63.2 (11.2)	57.5 (11.1)	**1.05 (1.02; 1.08); 0.001**	-	-
BRCA mutated (Ref. = wt)	10 (16.4)	62 (42.5)	**0.27 (0.13; 0.56); 0.001**	-	-
BMI at baseline	23.8 (21.2–27.2)	24 (21.6–27.7)	0.99 (0.93; 1.04); 0.632	0.96 (0.89; 1.04); 0.369	1.00 (0.89; 1.13); 0.978
Menopause	50 (82.0)	99 (67.8)	**2.16 (1.03; 4.52); 0.041**	0.75 (0.23; 2.37); 0.620	1.64 (0.25; 10.54); 0.602
Ca125	938.4 (350.4–2132)	858 (311–2135)	1.00 (0.99; 1.00); 0.776	1.00 (1.00; 1.00); 0.752	1.00 (1.00; 1.00); 0.297
Ascites	42 (68.8)	69 (47.3)	**2.47 (1.31; 4.64); 0.005**	1.71 (0.82; 3.55); 0.149	5.84 (0.61; 55.75); 0.125
Primary treatment (*Ref. Non cytoreduced*)					
*Non cytoreduced*	18 (29.5)	1 (0.7)	-	-	-
* PDS*	23 (37.7)	102 (69.9)	**0.01 (0.00; 0.10); <0.001**	**0.02 (0.00; 0.19); <0.001**	0.00 (0.00; Inf^); 0.989
* IDS*	20 (32.8)	43 (29.4)	**0.03 (0.00; 0.21); 0.001**	**0.04 (0.00; 0.36); 0.004**	0.00 (0.00; Inf^); 0.990
RT (ref = 0)					
0	35 (57.4)	127 (87.0)	-	-	-
* 1–10 mm*	8 (13.1)	13 (8.9)	*2.23 (0.86; 5.81); 0.099*	*3.24 (0.90; 11.74); 0.073*	0.58 (0.06; 5.26); 0.627
* >10 mm*	18 (29.5)	6 (4.1)	**10.88 (4.02; 29.50); <0.001**	**9.45 (2.86; 31.18); <0.001**	0.71 (0.06; 8.43); 0.788
**Molecular markers**					
Nucleus AR score	0 (0–1)	0 (0–2)	1.00 (0.85; 1.17); 0.987	0.92 (0.75; 1.14); 0.455	1.25 (0.89; 1.77); 0.197
PR score	1 (0–2)	1 (0–3)	**0.83 (0.71; 0.97); 0.019**	0.87 (0.73; 1.04); 0.137	0.87 (0.55; 1.38); 0.556
ERα score	4 (2–8)	4 (2–8)	1.06 (0.97; 1.15); 0.230	1.01 (0.92; 1.12); 0.793	1.11 (0.87; 1.42); 0.401
Nucleus ERβ1 score	4 (3–8)	5 (3–8)	0.98 (0.89; 1.09); 0.751	0.96 (0.86; 1.08); 0.512	0.96 (0.73; 1.25); 0.764
Cytoplasm ERβ1 score	2 (0–3)	3 (0–4)	0.94 (0.82; 1.07); 0.341	*0.86 (0.72; 1.03); 0.096*	1.22 (0.91; 1.65); 0.179
Nucleus ERβ2 score	6.7 (3.1)	7.2 (3.3)	0.95 (0.87; 1.04); 0.304	0.91 (0.82; 1.02); 0.111	1.08 (0.86; 1.36); 0.491
Cytoplasm ERβ2 score	2 (0–3)	2 (0–3)	0.93 (0.80; 1.08); 0.348	0.89 (0.75; 1.06); 0.197	1.15 (0.78; 1.70); 0.471
Nucleus ERβ5 score	6.2 (2.6)	6.2 (3.1)	1.00 (0.91; 1.11); 0.960	0.94 (0.83; 1.07); 0.356	*1.24 (0.96; 1.59); 0.096*
Cytoplasm ERβ5 score	0 (0–0)	0 (0–3)	0.88 (0.73; 1.06); 0.185	0.88 (0.71; 1.10); 0.264	1.10 (0.69; 1.74); 0.686
ERα/ERβ1nuc ratio	1 (0.4–2.2)	0.8 (0.4–1.5)	1.04 (0.91; 1.18); 0.591	0.99 (0.83; 1.17); 0.889	1.20 (0.89; 1.62); 0.227
ERα/ERβ2nuc ratio	0.8 (0.3–1.5)	0.6 (0.3–1.0)	1.06 (0.87; 1.29); 0.549	1.02 (0.82; 1.27); 0.853	1.27 (0.73; 2.21); 0.394
ERα/ERβ5nuc ratio	0.8 (0.3–1.3)	0.8 (0.3–1.3)	0.95 (0.80; 1.13); 0.533	0.93 (0.77; 1.13); 0.488	0.73 (0.31; 1.73); 0.472
P53 Status (*Ref. wt*)					
* Wt*	2 (3.3)	9 (6.2)	-	-	-
* Mutated null-type*	14 (23.0)	41 (28.1)	1.54 (0.30; 7.98); 0.609	1.80 (0.30; 10.68); 0.520	Inf^ (0.00; Inf^); 0.987
* Mutated overexpressed*	45 (73.8)	96 (65.7)	2.11 (0.44; 10.16); 0.352	2.10 (0.39; 11.27); 0.391	Inf^ (0.00; Inf^); 0.987

**Abbreviations:** wt: wild type; BMI: body mass index; IDS: interval debulking surgery; PDS: primary debulking surgery; RT: residual tumour; AR: androgen receptor; PR: progesterone receptor; ER: oestrogen receptor; OR: odds ratio; IOR: interaction odds ratio; 95%CI: 95% confidence interval; Ref.: reference; ^Inf: infinite (due to poor or null variability within predictors). * In **bold**: the significant results (*p* < 0.05), in *italics*: the suggestive results (0.05 < *p* < 0.10).

## Data Availability

The data presented in this study are available on request from the corresponding author. The data are not publicly available due to privacy issues.

## Appendix A. Additional Methods

### Appendix A.1. Data Collection

Histopathologic features were reviewed and collected in an electronic database, alongside epidemiologic, clinical, and surgical data.

In depth, among clinical data we recorded age, body mass index (BMI), menopausal status, comorbidities (e.g., ascites), previous surgical treatment, BRCA mutational status (wild-type BRCA1, BRCA2), pre-operative CA125. We further reported clinical stage, according to the criteria of the International Federation of Gynaecology and Obstetrics (FIGO), and primary treatment (either interval or primary debulking surgery), and platinum resistance. Finally, data related to follow-up status, i.e., overall survival (OS) were further collected.

### Appendix A.2. Immunohistochemistry

Formalin-fixed, paraffin-embedded tissue sections were deparaffinized and subjected to antigen retrieval using low/high pH Target Retrieval Solution (Agilent Technologies, Santa Clara, CA, USA) in the DAKO PT Link module (Agilent Technologies). Antibodies used include anti-ERα (Clone SP1, Invitrogen, Thermo Fisher Scientific, Walthman, MA, USA, 1:200), anti-ERβ1 (clone PPG5/10, Bio-Rad, Bio-Rad Laboratories, Hercules, CA, USA, dilution 1:50), anti-ERβ2 (clone 57/3, Bio-Rad, dilution 1:100), anti-ERβ5 (clone 5/25 Bio-Rad, dilution 1:100), anti-PR (clone 1E2, Ventana, Ventana Medical Systems, Inc., Tucson, AZ, USA, prediluted), and anti-AR (clone AR441, Dako, dilution 1:50). Slides were counterstained with Mayer’s haematoxylin, dehydrated in ethanol and xylene, and finally mounted. Staining without primary antibody was used to validate secondary antiserum specificity, while a section from a tissue known to express the protein of interest was used as a positive control. Antibodies used were previously validated by us and other groups and widely used in clinical studies for detection in paraffin-embedded tissue sections [40,41,42].

### Appendix A.3. Complete Statistical Analysis

We finally enrolled 207 women, 65.2% of whom were BRCA-wt. Given the retrospective nature of the study, no prior sample size calculation was available. However, such sample size is able to achieve a 80% power to detect a difference of 0.4 using a two-sided Mann–Whitney U test assuming a normal data distribution, a significance level (alpha) of 0.050, and standard deviation of 1.0 in both groups. Power analysis was conducted with PASS2021 [20].

The whole data were preliminarily summarized by descriptive statistics, both on the overall sample and according to BRCA mutation status, i.e., wild-type vs. BRCA1/2. In depth, qualitative data were described as absolute and relative percentage frequencies. The Gaussian distribution of quantitative variables was assessed by the Shapiro–Wilk test and data expressed either as mean and standard deviation (SD) or median and interquartile range (IQR). Between-group differences on qualitative data were computed by either the chi-square test or Fisher–Freeman–Halton’s exact test, as appropriate. Quantitative variables were instead assessed either by Student’s *t* test or the Mann–Witney U test. Missing values in quantitative variables, all <5%, were treated by multiple imputation with lasso regression methods centred on the mean by the *imputeR* R package [21]. Differences across BRCA mutational status, classified as “wild-type”, “BRCA1”, and “BRCA2” mutated, stratified for menopause status, were assessed by the Kruskal–Wallis non-parametric test. Pairwise comparisons were assessed by Dunn’s test, with FdR correction for multiple comparisons (i.e., false discovery rate). The whole data were further presented by “violin plots” drawn with R packages “*ggpubr*”, “*ggplot2*”, and “*ggstatsplot*” [43,44,45].

To evaluate the raw effects of each molecular marker and clinical data (predictor) on OS, ordinary proportional hazard Cox models were fitted, and hazard ratios (HRs) and 95% confidence intervals (CIs) reported. To evaluate combined effects between molecular markers/clinical predictors and BRCA mutations, multivariable age-adjusted interaction Cox models were fitted, one for each predictor, and the related interaction HRs (IHR) reported. In this framework, IHR = 1 indicated no synergy between predictor and BRCA mutation, IHR < 1 expressed a reduction in hazard due to the synergy, whilst IHR > 1 denoted an increased hazard. In summary, the coefficients of the main effects (in exponential terms) were interpreted as HRs of the outcome by considering a unit increase in the molecular marker in the BRCA-wt or an increase in the hazard of the outcome occurring, as compared with the (arbitrarily) chosen reference group for categorical predictors (HRpredictor). IHRs were interpreted as difference (in HR terms) of predictor variations between BRCA conditions (with wild-type as reference category). Proportionality of the hazard functions was assessed by visual inspection of hazard plots and Schoenfeld residuals. When proportionality was doubtful, weighted Cox regression models were fitted [46,47].

Potential predictors of platinum resistance were instead assessed by logistic regression models. To evaluate the combined effects between hormone receptor expression (HRE)/clinical data and BRCA mutations, multivariable interaction models were fitted, one per each predictor, and the interaction odds ratios (IOR) reported. In summary, the coefficients of the main effects (in exponential terms) were interpreted as ORs of the outcome by considering a unit increase in the predictor in the wtBRCA (ORpredictor) as for quantitative predictors, and as an increase in the odds of the outcome occurring, compared with the (arbitrarily) chosen reference group for qualitative data. The interaction parameters (IOR) were interpreted as difference (in OR terms) of predictor variations between BRCA conditions (wild-type as reference category). Multivariable interaction models were applied in place of classic multivariable models in order to better assess the role of each predictor on clinical outcomes according to BRCA mutational status.

In the case of null score values, <5% pseudo counts were arbitrarily chosen at 0.5 to assess ERα/ERβs ratios. In the case of higher percentages of null score values, such as for AR and PR, characterized by a largely asymmetric distribution, this was not possible. In fact, the use of pseudo-counts to make all observed counts strictly positive, applied for ERα/ERβs ratios, would have in that case provided dramatically biased estimates, given over 50% of women presented a null AR score (i.e., negative) and almost 40% a null PR score. In the case of high-throughput data such as RNA-seq experiments, we might have been able to apply inferential methods to extract appropriate pseudocounts [39]. Statistical significance was set at *p* value < 0.05. P-values between 0.05 and 0.10 were also reported as suggestive. All analyses were performed by using R software version 4.2.0 (CRAN ^®^, R Core Team, 2022, Vienna, Austria) [22], and its packages *Hmisc*, *survival*, *survminer*, and *coxphw* [48,49,50,51,52].
